# The effects of resistance exercise snacks via a mobile health app on musculoskeletal strength in sedentary young women: a randomized controlled trial

**DOI:** 10.3389/fphys.2026.1926054

**Published:** 2026-09-30

**Authors:** Zhangyuting Shang, Ziren Zhao, Xin Zheng, Fangyuan Zhao, Yan Ma, Zheng Liu, Kaixiang Zhou

**Affiliations:** 1 Sports Coaching College, Beijing Sport University, Beijing, China; 2 College of Physical Education and Health Management, Chongqing University of Education, Chongqing, China; 3 College of Physical Education and Health Science, Chongqing Normal University, Chongqing, China; 4 School of General Education, Chongqing Jianzhu College, Chongqing, China; 5 Department of Rehabilitation, University-Town Hospital of Chongqing Medical University, Chongqing, China

**Keywords:** exercise snacks, explosive strength, maximal strength, muscular endurance, sedentary women

## Abstract

**Objective:**

This study aims to evaluate the effects of an eight-week Exercise Snacks (ES) program based on a mobile app on musculoskeletal strength in sedentary young women.

**Methods:**

Sixty sedentary young women were randomly assigned to the ES group (n=30) or the control group (n=30). Participants in the ES group completed an eight-week exercise program with 75 minutes per week. Outcome measures before and after intervention included maximal strength (peak torque, maximal force output), explosive strength (standing long jump), and muscular endurance (total work at 180°/s, bodyweight squats to failure). We used two-way repeated measures analysis of variance (ANOVA) (time × group) with Bonferroni *post hoc* tests, and effect sizes were reported as partial eta squared ( ${\text{η}}_{p}^{2}$). p < 0.05 was statistically significant.

**Results:**

Significant group × time interactions were observed for all outcomes (p < 0.05). In the ES group, peak torque at 60°/s increased from 115.68 ± 33.92 N·m to 128.40 ± 36.88 N·m, while the control group showed a change from 116.76 ± 30.32 N·m to 118.85 ± 30.20 N·m (p = 0.011, $\eta_{p}^{2}$ = 0.107). Maximal force output in the ES group increased from 568.07 ± 43.45 N to 579.61 ± 46.35 N, compared to the control group, which decreased from 558.98 ± 44.26 N to 557.46 ± 45.16 N (p = 0.026, $\eta_{p}^{2}\;$= 0.083). The standing long jump improved from 165.20 ± 10.45 cm to 172.17 ± 10.72 cm in the ES group, whereas the control group improved from 167.27 ± 9.98 cm to 168.43 ± 9.74 cm (p < 0.001, $\eta_{p}^{2}\;$= 0.258). Total work at 180°/s in the ES group increased from 722.82 ± 109.39 J to 812.85 ± 128.35 J, while the control group showed an increase from 738.86 ± 81.02 J to 760.73 ± 79.80 J (p < 0.001, $\eta_{p}^{2}$ = 0.285). Lastly, the number of bodyweight squat repetitions increased from 27.87 ± 9.66 to 32.53 ± 10.88 in the ES group, compared to the control group, which increased from 25.67 ± 7.22 to 27.73 ± 7.90 (p = 0.019, $\eta_{p}^{2}$= 0.091).

**Conclusions:**

75 minutes of resistance exercise snacks per week is an effective strategy for promoting musculoskeletal strength in sedentary women. Further studies should explore the underlying physiological mechanisms.

## 1 Introduction

Musculoskeletal strength is a fundamental component of physical fitness and is essential for musculoskeletal function and metabolic health. Sedentary behavior (SB) characterized by prolonged periods of sitting with minimal skeletal muscle contractile activity (Tremblay et al., 2017; Bull et al., 2020; Dempsey et al., 2020; World Health Organization, 2022), reduces opportunities for habitual mechanical loading and may adversely affect musculoskeletal strength. A growing body of evidence has shown that SB is associated with cardiovascular disease, type 2 diabetes, and increased all-cause mortality (Ekelund et al., 2016; Patterson et al., 2018; Bull et al., 2020). Epidemiological study further indicated a dose–response relationship between sedentary time and mortality risk, with the risk of all-cause mortality increasing progressively to approximately 1.48-fold at 10 h/day and 2.92-fold at 12 h/day (Ekelund et al., 2019). Prolonged sedentary time has also been linked to muscle atrophy, and functional impairments in the lower back, gluteal muscles, and lower limbs (García-Hermoso et al., 2018; Li et al., 2018; Boukabache et al., 2021; Kett et al., 2021; Ramsey et al., 2021; Gilchrist et al., 2022). These adverse musculoskeletal effects may be especially relevant to women, who are more significantly impacted by insufficient physical activity: in 2022, the global prevalence was 33.8% among women compared to 28.7% among men (Strain et al., 2024).This trend is similarly observed among university students, where 69% of women sit for over 6 hours a day, versus 46% of men (Vainshelboim et al., 2019). In addition, women typically have lower skeletal muscle mass, averaging 21.0 kg compared to 33.0 kg in men, which may impact muscle maintenance during prolonged sitting (Janssen et al., 2000). Importantly, declining musculoskeletal strength in women has been associated with a higher risk of all-cause mortality (García-Hermoso et al., 2018; LaMonte et al., 2026). Due to the high rates of inactivity, prolonged sedentary behavior, and lower muscle strength among women, there is a clear need to evaluate accessible strength-promoting interventions tailored for this group.

Regular physical activity is widely recommended as an effective strategy to reduce health risks associated with SB (Thivel et al., 2018; Lavie et al., 2019; Stamatakis et al., 2019; Ekelund et al., 2020). Accordingly, the World Health Organization (WHO) recommends that adults accumulate 150–300 minutes of moderate-intensity physical activity or 75–150 minutes of vigorous-intensity physical activity per week, in addition to engaging in strength training involving all major muscle groups on at least two days per week (Bull et al., 2020). However, real-world adherence remains suboptimal, with global estimates indicating that approximately 31% of adults do not meet the recommended physical activity guidelines (World Health Organization, 2022; Strain et al., 2024; World Health Organization, 2024). Insufficient time has consistently been recognized as one of the most prominent barriers to regular exercise participation (Centers for Disease Control and Prevention, 2025). Therefore, there is a pressing need to identify time-efficient and accessible exercise strategies.

Exercise snacks (ES), defined as multiple short bouts of vigorous-intensity exercise performed throughout the day, has emerged as a time-efficient alternative to longer, continuous exercise sessions (Jones et al., 2024; Monteiro et al., 2025). By reducing the time required for each bout, ES may make physical activity easier to incorporate into daily routines, particularly among inactive individuals. A 6-week ES intervention was shown to be highly acceptable and achievable among inactive young adults (Yin et al., 2025). Although previous studies have demonstrated beneficial effects of ES on cardiometabolic health and body composition (Islam et al., 2022; Wan et al., 2025), evidence regarding its effects on musculoskeletal strength remains limited and is largely derived from small pilot studies (Perkin et al., 2019; Fyfe et al., 2022; Brandt et al., 2024; Rodríguez et al., 2026). A 28-day unsupervised home-based ES significantly improved 60-second sit-to-stand in older adults (Perkin et al., 2019). In contrast, Fyfe et al. reported that a 4-week home-based ES could not enhance 30-second sit-to-stand in older adults (Fyfe et al., 2022). This inconsistency may stem from a small sample size or insufficient training volume. Therefore, further research should examine whether ES can effectively enhance musculoskeletal strength, especially in real-world settings.

However, implementation of ES requires participants to complete multiple brief exercise bouts throughout the day. In this context, insufficient guidance, reminders, or supervision may affect exercise quality and adherence to the prescribed protocol (Yin et al., 2025). Mobile health applications (App) may provide a practical way to support ES delivery by offering standardized exercise instructions and automated reminders. Recent evidence suggests that app-based ES interventions are feasible and acceptable in real-world settings and may achieve high adherence (Fyfe et al., 2022; Hu et al., 2026; Rodríguez et al., 2026). For instance, Babir et al. found that a remotely delivered ES was feasible and achieved high adherence among inactive adults (Babir et al., 2025). Collectively, these findings suggest that mobile health app-based ES may be a practical strategy for real-world implementation. In the present study, the mobile app was used primarily to deliver and support the ES intervention rather than as an independent intervention component.

Accordingly, this study aims to evaluate the effects of an eight-week ES intervention delivered via a mobile health app on musculoskeletal strength in sedentary young women. We hypothesized that, compared with the control group, participants in the ES group would demonstrate significantly greater improvements in maximal strength, explosive strength, and muscular endurance.

## 2 Materials and methods

### 2.1 Participants

The sample size was estimated to use G*Power with reference to Wong et al. (2024). Based on repeated-measures ANOVA design (group × time interaction), with an alpha level of 0.05, power of 0.80, and an expected medium effect size, a total sample of 40 participants was required. Sixty participants were recruited for this study. The inclusion criteria were: (1) women aged 18–25 years; (2) an average daily sedentary time of at least 8 hours, as determined by the Sedentary Behavior Questionnaire (SBQ); (3) less than 75 minutes of vigorous-intensity physical activity or less than 150 minutes of moderate-intensity physical activity per week, as assessed by the International Physical Activity Questionnaire (IPAQ); and (4) regular menstrual cycles without the use of hormonal contraceptives. The exclusion criteria were: (1) risk of participating in physical activity based on the Physical Activity Readiness Questionnaire (PAR-Q); and (2) smoking or drug use. The study was approved by the Ethics Committee of the University-Town Hospital of Chongqing Medical University (IIT 2025-077), and all procedures were performed in accordance with the Declaration of Helsinki. The trial was retrospectively registered in the Medical Research Registration Information System (PID 318652) on 14/04/2026.

### 2.2 Study design

This study used a single-blind (assessor-blind) randomized controlled trial (**Figure 1**). We randomly assigned participants to the ES group (n = 30) or the control group (n = 30) using a computer-generated randomization sequence. We utilized the mobile health App (Tizhi Cloud) to supervise the ES protocol. The App included standardized bodyweight exercises for squats, jump jacks, wall sits, squat jumps, and lunges. Each exercise lasts one minute. Participants received instruction on app operations and proper exercise techniques before the experiment. During the intervention period, participants interactively completed exercises guided by the App’s built-in videos. The app recorded their performance and assigned ratings, requiring an “S” rating (on a scale of S > A > B > C > D) to ensure proper training intensity after each session. It also recorded participants’ perceived exertion ratings (RPE) using Borg’s CR-10 scale, enabling remote monitoring.

**Figure 1:**
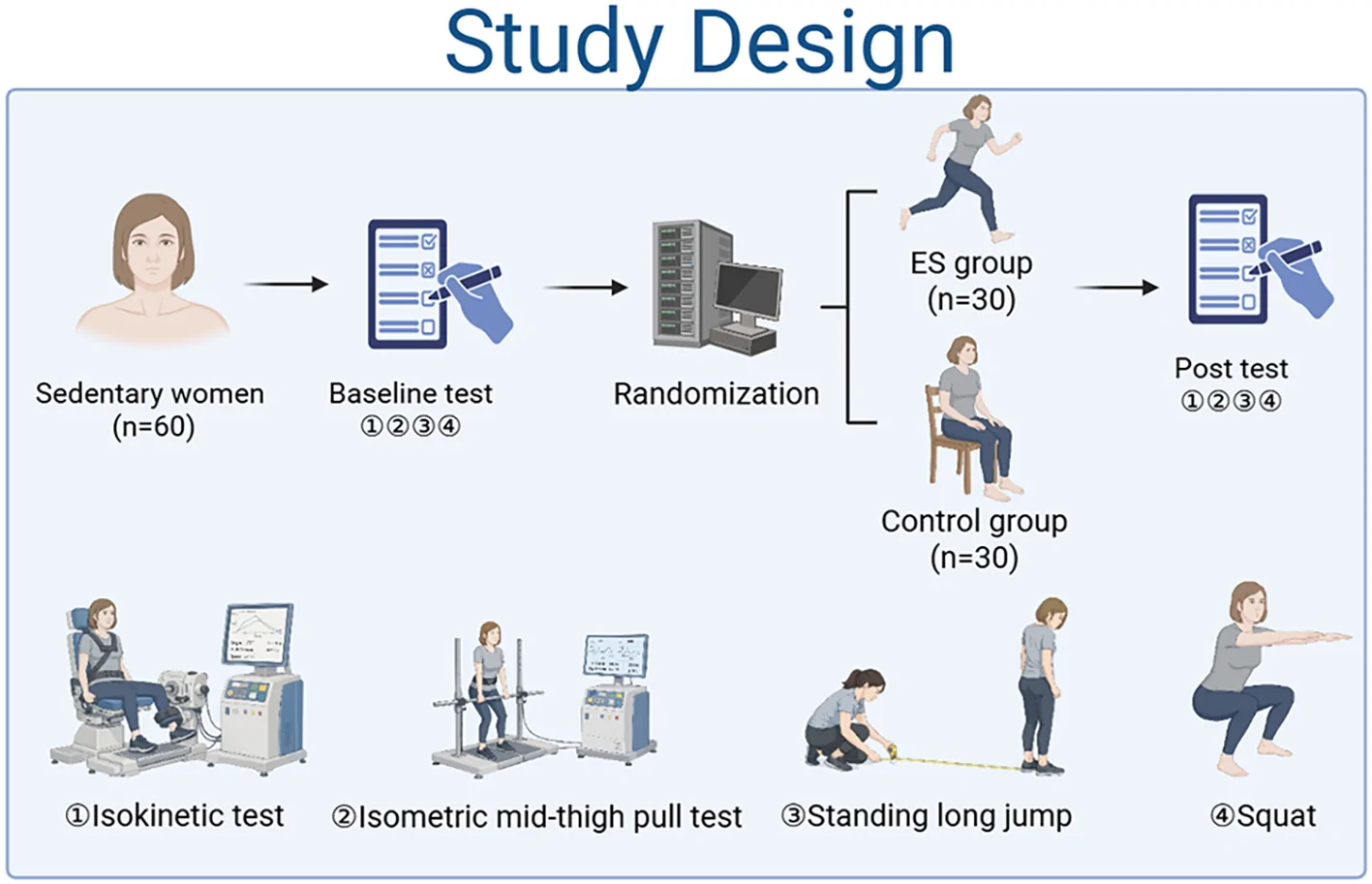
Study design.

#### 2.2.1 Exercise protocol

Participants in the ES group exercised 7 days per week, with 11 bouts from Monday to Friday and 10 bouts on weekends. Each exercise bout was performed continuously for 1 minute, with participants required to achieve an RPE of ≥ 7 on Borg’s CR-10 scale. Consecutive exercise bouts were separated by 30 minutes (**Figure 2**). This distribution was designed primarily to accumulate the prescribed exercise volume through multiple brief bouts rather than through a continuous exercise session. The 30-minute interval kept consecutive bouts separate and avoided clustering multiple bouts into a single continuous exercise session. Participants in the control group maintained their habitual lifestyle. All testing sessions were conducted at the same time of day for each participant. During menstruation, participants could suspend training if they were uncomfortable. We recorded suspension time and redistributed it the following week. Adherence was calculated as the number of exercise bouts completed each day divided by the number of bouts prescribed for that day × 100%. An exercise bout was recorded as completed only when it met all prescribed criteria, including an exercise duration of 1 minute, an RPE of ≥7, and a 30-minute interval from the preceding bout. We verified completion using records from the mobile app.

**Figure 2:**
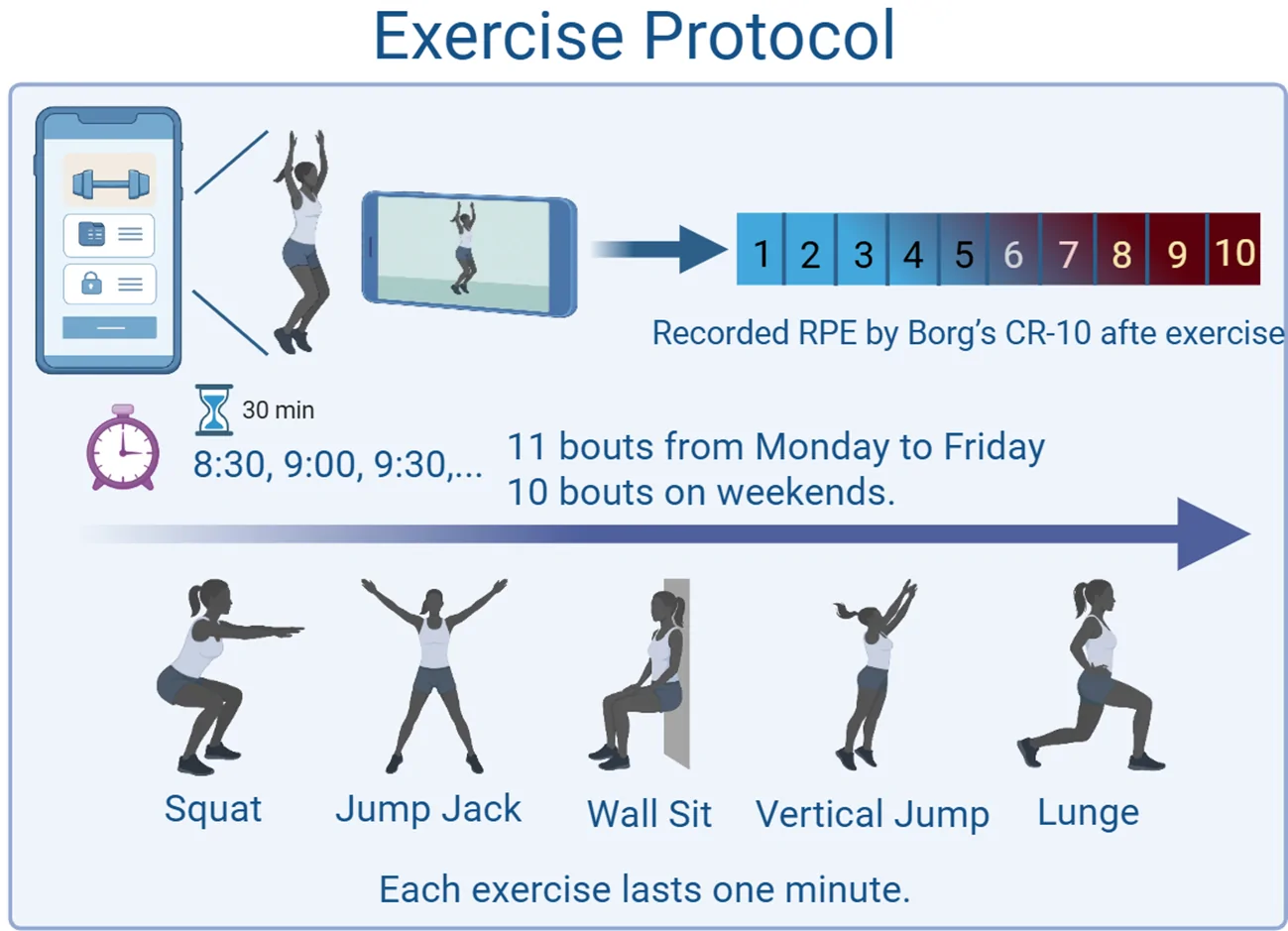
Exercise protocol.

#### 2.2.2 Outcomes measurement

##### 2.2.2.1 Isokinetic test

We assessed knee extensor strength using an isokinetic dynamometer (IsoMed 2000, D&R Ferstl GMBH, Hemau, Germany). Participants sat with their hips at 90°flexion; the dynamometer axis was aligned with the lateral femoral epicondyle, and the trunk, pelvis, tested thigh, and non-tested lower leg were secured (Obara et al., 2023). After a 5-minute jog warm-up, participants performed concentric knee extension and flexion through a range of motion from 10° knee extension to 90° knee flexion. Gravity correction was applied to minimize the effects of the lower leg and dynamometer arm weights on the results. The testing involved five repetitions at 60°/s and fifteen at 180°/s. The highest values for peak torque (N m) at 60°/s and total work (J) at 180°/s across attempts were recorded for analysis.

##### 2.2.2.2 Isometric mid-thigh pull test

We used the isometric mid-thigh pull test (IMTP) to assess the multi-joint strength of the participants. Participants stood on a force plate (Chronojump Boscosystem, ≥1000Hz, Spina) with feet shoulder-width apart and positioned the bar at mid-thigh level. Researchers adjusted the knee and hip angles to 130° and 145° using a handheld goniometer. Participants were instructed to gradually tense their muscles, then pull as forcefully and rapidly as possible for 3–5 seconds, maintaining a rigid body posture and full foot contact. Verbal encouragement was provided throughout to promote maximum effort. Each participant conducted three maximal attempts with a 2-minute rest interval between trials. The highest maximal force output (N) across attempts was recorded for analysis.

##### 2.2.2.3 Standing long jump

The standing long jump (SLJ) test was used to assess lower-limb explosive strength. Participants stood behind a marked starting line with their feet approximately shoulder-width apart and their arms freely at their sides. From a stationary position, participants performed a countermovement with their arms and legs, then jumped as far as possible. Participants were instructed to land with both feet simultaneously and maintain balance without falling backward or taking additional steps. The horizontal distance from the starting line to the posterior heel of the rear foot at landing was measured using a tape measure to the nearest 1 cm. Each participant completed three maximal attempts with a one-minute rest interval between trials, and the longest distance was recorded for analysis.

##### 2.2.2.4 Bodyweight squats to failure

We evaluated lower-limb muscular endurance using a standardized bodyweight squat test at a steady pace of 2/0/2/0 (2 seconds lowering, no pause at the bottom, 2 seconds rising, no pause at the top). Participants stood with shoulder-width apart, toes pointing forward, and arms crossed over the chest to reduce upper limb assistance (Wilk et al., 2021). Each squat involved lowering until the thighs were parallel to the floor, then rising to full knee and hip extension, keeping heels on the ground and back upright (Escamilla et al., 1998). The test ended if participants couldn’t maintain tempo or depth for two repetitions. The total count of correctly performed repetitions was recorded for analysis.

### 2.3 Statistical analysis

We calculate the percentage change (Δ%) for each outcome. All data as mean ± standard deviation (SD) and assessed normality using the Shapiro–Wilk test. The independent T-test was used to compare participant baseline characteristics between the ES and control groups (**Table 1**). An analysis of outcomes was conducted using a two-way (time × group) repeated measures ANOVA, followed by *post hoc* tests with the Bonferroni correction. Effect sizes were expressed as partial eta-squared ( ${\text{η}}_{p}^{2}$), with effects categorized as trivial ( ${\text{η}}_{p}^{2}$ < 0.01), small (0.01 ≤ $\;{\text{η}}_{p}^{2}$ ≤ 0.06), moderate (0.06 ≤ ${\text{η}}_{p}^{2}$ ≤ 0.14), and large ( ${\text{η}}_{p}^{2}$ ≥ 0.14) (Cohen, 2013; Hill et al., 2024). p < 0.05 was considered statistically significant. All analyses were performed with SPSS version 27.0 (IBM Statistics, Chicago, IL).

**Table 1:** Baseline participant characteristics by groups.

Variable	ES (n=30)	CON (n=30)	*p^#^*
Body mass (kg)	63.64 ± 10.07	62.47 ± 12.34	*0.690*
Prak torque at 60°/s (N·m)	115.68 ± 33.92	116.76 ± 30.32	*0.896*
Maximal force output (N)	568.07 ± 43.45	558.98 ± 44.26	*0.425*
Standing long jump (cm)	165.20 ± 10.45	167.27 ± 9.98	*0.437*
Total work at 180°/s (J)	722.82 ± 109.39	738.86 ± 81.02	*0.737*
Squat	27.87 ± 9.66	25.67 ± 7.22	*0.322*

#: The p-value obtained using an independent samples t-test.

## 3 Results

We recruited 98 participants, of whom 60 were ultimately included. All 60 randomized participants completed the 8-week intervention, with no withdrawals or loss to follow-up; adherence to the prescribed ES intervention was 100%.

### 3.1 Maximal strength

#### 3.1.1 Peak torque at 60°/s (single joint)

A significant group × time interaction was observed (F = 6.917, p = 0.011, ${\text{η}}_{p}^{2}$ = 0.107, moderate effect). The main effect of time showed a significant difference (F = 13.425, p < 0.001, ${\text{η}}_{p}^{2}$ = 0.188, large effect). The main effect of group showed a non-significant difference (F = 0.262, p = 0.611, ${\text{η}}_{p}^{2}$ = 0.005, trivial effect) (**Figure 3A**).

**Figure 3:**
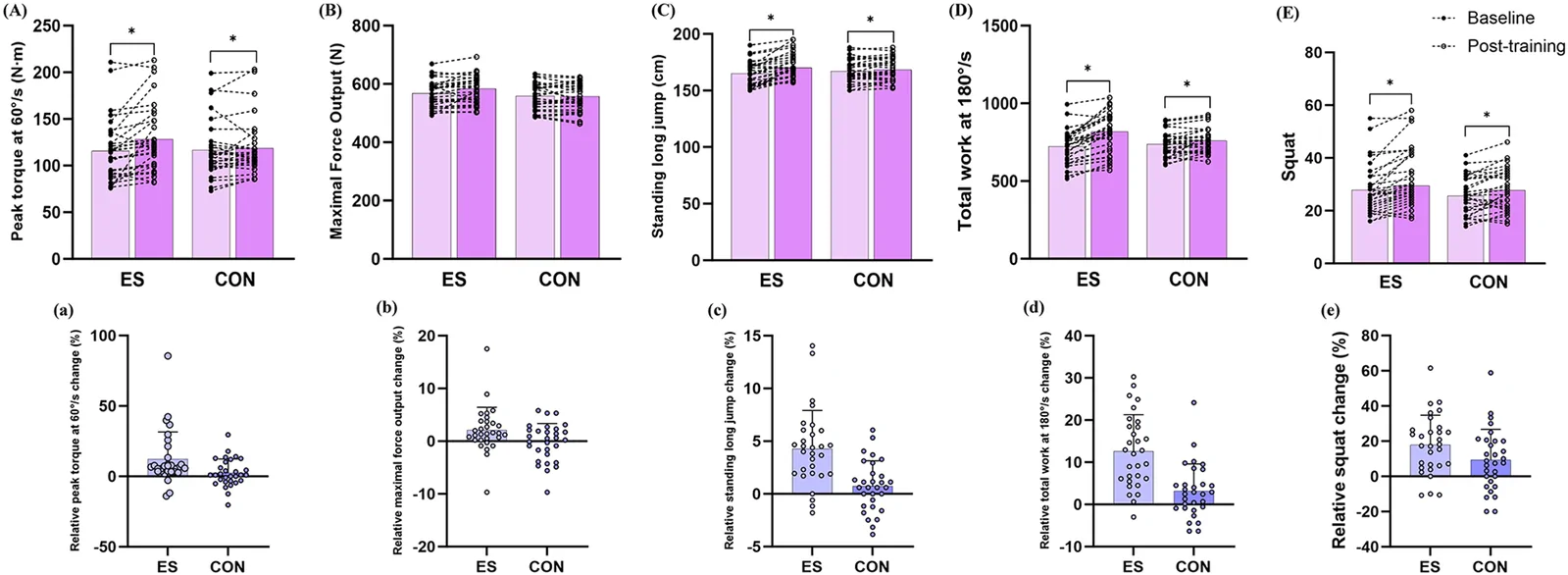
The effects of ES on musculoskeletal strength. Upper panels: **(A)** peak torque at 60°/s, **(B)** maximal force output, **(C)** standing long jump, **(D)** total work at 180°/s, and **(E)** squat. Lower panels: **(a)** relative peak torque at 60°/s change (%), **(b)** relative maximal force output change (%), **(c)** relative standing long jump change (%), **(d)** relative total work at 180°/s change (%), and **(e)** relative squat change (%). *p < 0.05 indicates a significant within-group difference between baseline and post-training based on Bonferroni-adjusted *post hoc* comparisons.

The ES group increased by 12.34% (from 115.68 to 128.40 Nm), compared to 2.54% (from 116.76 to 118.85 Nm) in the control group (**Figure 3a**; **Table 2**).

**Table 2:** The effects of the ES group and CON group on the musculoskeletal.

Group	Pre	Post	Δ% (Mean)	*p^#^*			
				Time	Group		Interaction
Prak torque at 60°/s (N·m)							
ES	115.68 ± 33.92	128.40 ± 36.88	12.34	*<0.001*		*0.611*	*0.011*
CON	116.76 ± 30.32	118.85 ± 30.20	2.54				
Maximal force output (N)							
ES	568.07 ± 43.45	579.61 ± 46.35	2.09	*0.084*		*0.169*	*0.026*
CON	558.98 ± 44.26	557.46 ± 45.16	-0.23				
Standing long jump (cm)							
ES	165.20 ± 10.45	172.17 ± 10.72	4.29	*<0.001*		*0.746*	*<0.001*
CON	167.27 ± 9.98	168.43 ± 9.74	0.74				
Total work at 180°/s (J)							
ES	722.82 ± 109.39	812.85 ± 128.35	12.61	*<0.001*		*0.478*	*<0.001*
CON	738.86 ± 81.02	760.73 ± 79.80	3.20				
Squat							
ES	27.87 ± 9.66	32.53 ± 10.88	17.92	*<0.001*		*0.128*	*0.019*
CON	25.67 ± 7.22	27.73 ± 7.90	9.34				

#The p-value obtained using a two-way repeated measures ANOVA.

#### 3.1.2 Maximum force in IMTP (multi joint)

A significant group × time interaction was observed (F = 5.253, p =0.026, ${\text{η}}_{p}^{2}$ =0.083, moderate effect). The main effect of time showed a non-significant difference (F = 3.096, p = 0.084, ${\text{η}}_{p}^{2}$ = 0.051, small effect). The main effect of group showed a non-significant difference (F = 1.941, p =0.169, ${\text{η}}_{p}^{2}$ =0.032, small effect) (**Figure 3B**).

The ES group increased by 2.09% (from 568.07 to 579.61 N), compared to -0.23% (from 558.98 to 557.46 N) in the control group (**Figure 3b**).

### 3.2 Explosive strength

#### 3.2.1 Standing long jump

A significant group × time interaction was observed (F = 20.196, p < 0.001, ${\text{η}}_{p}^{2}$ = 0.258, large effect). The main effect of time showed a significant difference (F = 39.714, p < 0.001, ${\text{η}}_{p}^{2}$ = 0.406, large effect). The main effect of group showed a non-significant difference (F = 0.106, p = 0.746, ${\text{η}}_{p}^{2}$ = 0.002, trivial effect) (**Figure 3C**).

The ES group increased by 4.29% (from 165.20 to 172.17 cm), compared to 0.74% (from 167.27 to 168.43 cm) in the control group (**Figure 3c**).

### 3.3 Muscular endurance

#### 3.3.1 Total work at 180°/s (single joint)

A significant group × time interaction was observed (F = 23.145, p < 0.001, ${\text{η}}_{p}^{2}$ = 0.285, large effect). The main effect of time showed a significant difference (F = 62.367, p < 0.001, ${\text{η}}_{p}^{2}$ = 0.518, large effect). The main effect of group showed a non-significant difference (F = 0.509, p = 0.478, ${\text{η}}_{p}^{2}$ = 0.009, trivial effect) (**Figure 3D**).

The ES group increased by 12.61% (from 722.82 to 812.85 J), compared to 3.20% (from 738.86 to 760.73 J) in the control group (**Figure 3d**).

#### 3.3.2 Bodyweight squats to failure (multi joint)

A significant group × time interaction was observed (F = 5.820, p = 0.019, ${\text{η}}_{p}^{2}$ = 0.091, moderate effect). The main effect of time showed a significant difference (F = 39.033, p < 0.001, ${\text{η}}_{p}^{2}$ = 0.402, large effect). The main effect of group showed a non-significant difference (F = 2.379, p = 0.128, ${\text{η}}_{p}^{2}$ = 0.039, small effect) (**Figure 3E**).

The ES group increased by 17.92% (from 27.87 to 32.53), compared to 9.34% (from 25.67 to 27.73) in the control group (**Figure 3e**).

## 4 Discussion

This is the first study to investigate the effects of ES via a mobile app on musculoskeletal strength in sedentary young women. The findings show that, compared with the control group, ES could increase maximal strength, explosive strength, and muscular endurance in sedentary young women. These findings support that the ES as a practical strategy for improving musculoskeletal strength in sedentary populations.

The improvement in maximal strength was consistent with previous studies. For instance, a 6-week blood flow restricted staircase sprint ES increased peak torque in physically inactive college students (Zhu et al., 2025). Similarly, Perkin et al. reported that 28-day resistance ES produced positive changes in leg press strength among inactive older adults (Perkin et al., 2019). In contrast, Brandt et al. reported that a 12-week resistance ES could not enhance maximal strength in sedentary female workers (Brandt et al., 2024). A 6-week study also found that all-out sprint ES could not improve peak torque in physically active adults (Wong et al., 2024). This discrepancy may stem from the participants’ physical activity levels and the exercise protocols. ES may be more beneficial for participants with low physical activity levels in maintaining or improving muscle strength (Cannon and Marino, 2010; Schoenell et al., 2016). Wong et al. found that the benefits of ES in improving muscle strength in physically active adults are limited (Wong et al., 2024), likely because these participants already have high muscle strength. Liang et al. conducted a 12-week ES protocol that included two 10-minute sessions each day (Liang et al., 2024). This protocol combined progressive resistance exercises with Tai Chi, focusing more on improving mobility and balance rather than maximizing strength development. Brandt et al. implemented a 12-week resistance-based electrical stimulation (ES) protocol that involved 10-minute sessions conducted five days a week (Brandt et al., 2024). During these sessions, participants performed bodyweight strength and mobility exercises consecutively for 30 to 60 seconds without resting between exercises. However, this protocol may have led to increased muscular fatigue and compromised movement quality. Additionally, the lack of full supervision could have further reduced exercise standardization and the consistency of the training stimulus. This study likely provided a greater stimulus for the lower limbs, as the protocol included repeated exercises aimed at promoting muscle strength development. The exercises were conducted in separate sessions throughout the day, which likely reduced muscle fatigue and allowed participants to perform at a higher quality.

App-based supervision may partly explain the positive outcomes observed in this study. The mobile health App offered standardized exercise videos, scheduled reminders, and automatic recording of exercise completion and RPE. Previous systematic reviews suggest that mobile apps and activity trackers may support physical activity participation, and features such as reminders, self-monitoring, and personalized feedback may contribute to user engagement and adherence (Szinay et al., 2020; Laranjo et al., 2021). However, the present study was not designed to isolate the mobile app’s independent effect. In addition, we did not collect self-reported or qualitative data on app usability, perceived usefulness, motivation, enjoyment, or barriers to participation. Therefore, although the app may have facilitated standardized intervention delivery and remote monitoring, the present study cannot determine its specific contribution to adherence or the observed improvements in musculoskeletal strength. Future studies could directly compare app-supported and non-app-supported ES interventions and include process evaluations to clarify the role of specific digital features.

Another important finding is that ES could improve explosive strength in sedentary young women (Othman et al., 2022; Peng et al., 2023). The improvement in explosive strength may be attributed to the increase in maximal strength observed in this study, since greater strength can provide a more stable foundation for rapid force development. Additionally, explosive strength is highly sensitive to early neuromuscular adaptations, especially in sedentary participants (Maffiuletti et al., 2016; Barranco-Ruiz and Villa-González, 2020). Repeated exposure to the plyometric exercise, such as squat jumps and jump jacks may enhance motor-unit recruitment, intermuscular coordination, and force-transmission efficiency, thereby improving the ability to generate force rapidly during explosive activities (Sale, 1988; Davies et al., 2015; Maffiuletti et al., 2016; Hirayama et al., 2017). One unanticipated result was that ES could enhance muscular endurance in sedentary young women A plausible explanation is that repeated bouts may repeatedly activate the muscle, delivering frequent neuromuscular stimulation that enhances fatigue resistance (Aagaard, 2003; Folland and Williams, 2007; Burgomaster et al., 2008; Little et al., 2010; Gibala et al., 2012; Del Vecchio et al., 2019). These improvements may be meaningful for the inactive population (e.g., older adults), as muscular endurance is important for daily activities, such as stair climbing and long walking.

## 5 Limitations

Several limitations should be considered when interpreting the findings of this study. This study focused on sedentary young women. Interpret findings on strength and endurance improvements within this group’s context, and avoid generalizing to men, older adults, or active individuals, as they may differ in baseline muscle strength, training status, and health status, which may affect their responses to ES. Second, while ES demonstrated improvements in musculoskeletal strength outcomes, no assessments regarding physiological, neuromuscular, or morphological changes were conducted. Future research should involve more diverse populations, longer intervention and follow-up periods, and mechanistic evaluations to more accurately determine the efficacy of ES, as well as the underlying mechanisms and practical implications.

## 6 Conclusions

In conclusion, an 8-week mobile app–delivered exercise snacks intervention performed for 75 minutes per week was effective in improving maximal strength, explosive strength, and muscular endurance in sedentary young women. Further studies are needed to confirm these findings in diverse populations and to explore the underlying physiological mechanisms.

## Data Availability

The raw data supporting the conclusions of this article will be made available by the authors, without undue reservation.
